# Determination of FRET orientation factor between artificial fluorophore and photosynthetic light-harvesting 2 complex (LH2)

**DOI:** 10.1038/s41598-022-19375-2

**Published:** 2022-09-05

**Authors:** Kazuhiro J. Fujimoto, Tomoya Miyashita, Takehisa Dewa, Takeshi Yanai

**Affiliations:** 1 Institute of Transformative Bio-Molecules (WPI-ITbM), Nagoya University, Furocho, Chikusa, Nagoya, 464-8601 Japan; 2 Department of Chemistry, Graduate School of Science, Nagoya University, Furocho, Chikusa, Nagoya, 464-8601 Japan; 3 Department of Life Science and Applied Chemistry, Graduate School of Engineering, Nagoya Institute of Technology, Gokiso-cho, Showa, Nagoya, 466-8555 Japan

**Keywords:** Physical chemistry, Theoretical chemistry

## Abstract

The orientation factor of fluorescence resonance energy transfer (FRET) between photosynthetic light-harvesting 2 complex (LH2) and artificial fluorophore (Alexa Fluor 647: A647) was theoretically investigated. The orientation factor of 2/3, i.e., the isotropic mean, is widely used to predict the donor–acceptor distance from FRET measurements. However, this approximation seems inappropriate because the movement of A647 is possibly restricted by the bifunctional linker binding to LH2. In this study, we performed molecular dynamics (MD) simulations and electronic coupling calculations on the LH2-A647 conjugate to analyze its orientation factor. The MD results showed that A647 keeps a position approximately 26 Å away from the bacteriochlorophyll (BChl) assembly in LH2. The effective orientation factor was extracted from the electronic coupling calculated using the transition charge from electrostatic potential (TrESP) method. With MD snapshots, an averaged orientation factor was predicted to be 1.55, significantly different from the isotropic mean value. The analysis also suggested that the value of the refractive index employed in the previous studies is not suitable for this system. Furthermore, optimal orientations of A647 with larger orientation factors to improve FRET efficiency were searched using Euler angles. The present approach is useful for extending the applicability of FRET analysis.

## Introduction

Fluorescence resonance energy transfer (FRET) is a phenomenon in which the excitation of acceptor molecules occurs simultaneously with the de-excitation of donor molecules, via transfer of energy to the acceptors^1–4^. With the development of various fluorescent proteins such as green fluorescent protein (GFP) mutants and the advancement of genetic engineering technology, the FRET phenomenon has been extensively used for bioimaging^5–8^. Whereas conventional biochemical approaches have difficulties in addressing the fundamental questions of when and where intracellular signal transduction occurs, FRET imaging technology has made it possible to visualize how gene products function dynamically in living cells^9^. With these advantages, FRET technology has now become an indispensable tool in molecular biology and medical science^10,11^.

The rate constant for FRET is derived from the so-called Förster theory of excitation-energy transfer (EET)^1–4^. It involves the six factors: (i) orientation factor between donor and acceptor, (ii) intermolecular distance between donor and acceptor, (iii) overlap between the fluorescence spectrum of the donor and the absorption spectrum of the acceptor, (iv) refractive index of the solvent, (v) fluorescence quantum yield of the donor, and (vi) fluorescence lifetime of the donor (see also Eq. (1) for details). Their relationship formulated in the Förster equation allows us to determine one of the six factors if the FRET rate constant is measured and the rest of the factors are somehow given. Based on this idea, structural analysis to determine the intermolecular distance between donor and acceptor has been conducted using the measured values of FRET rate constants^7,12,13^. However, there is an overlooked problem in this approach, which is about the orientation factor, denoted $\kappa^{2}$ in the Förster equation. The orientation factor arises from the so-called dipole–dipole (DD) approximation in Förster’s formalism, which is to treat the electronic coupling^14^ between the donor and acceptor molecules as a simplified DD interaction. It is a quantity determined by the direction and distance of the isolated donor-acceptor (DA) transition dipole moments, and ranges from 0 to 4^2,4,12,15^. For a practical reason, a constant 2/3 is widely used for $\kappa^{2}$ in any case, whereas the other factors of the Förster equation are provided by experimental or phenomenological means. It corresponds to an isotropic mean value derived by the further assumption that the transition dipole moments of donor and acceptor are randomly oriented in all directions, and rotate at rates higher than that of FRET^15,16^. This approximation to $\kappa^{2}$ is in fact considered to be usually rather satisfactory^15,16^. The 2/3 is valid for dynamic isotropic (random) averaging, and also there is a somewhat lower, static isotropic value of $\kappa^{2}$, which is 0.476^17,18^. However, in biological fluorescent systems, the movement of the donors and acceptors should be severely restricted in many cases, and thus their transition dipole moments are not likely to be fully reoriented randomly. This is due to the fact that the fluorescent dyes used as donors and acceptors are generally bound to the target protein by peptide linkers, thus implying that the isotropic mean value 2/3 could be inappropriate^19,20^. Therefore, the accuracy of the DA distance estimated from the widely-practiced FRET analysis based on the isotropic mean $\kappa^{2}$ is questionable, particularly for biological applications.

In order to validate the assumption $\kappa^{2}$ = 2/3, many studies have investigated the value of the orientation factor for realistic FRET systems in which the movement of the fluorophore is restricted^21–32^. In such studies, computational approaches involving molecular dynamics (MD) simulations play a vital role in analyzing the molecular orientation of fluorophores. VanBeek et al. performed MD simulations on a biological system in which a fluorescent dye was covalently bound to lysozyme via a succinimide linkage and analyzed their orientation factors^23^. The results showed that the orientation factors deviated significantly from 2/3 on average. A similar analysis based on the MD simulations was also performed by Dolghih et al.^27^, showing that shortening the length of the linker between the chromophores led to an increasingly larger shift in $\kappa^{2}$ from 2/3. It can thus be said that with these systems, the average DA orientation has anisotropic nature due to the influence of the linker; therefore, the widely used approximation $\kappa^{2}$ = 2/3 seems to be no longer reliable for these FRET analyses.

As written above, the orientation factor originates with the DD approximation to the calculation of electronic coupling. Our attention turns towards the validity of the underlying DD approximation for the FRET analysis. Its reliability should affect the characterization of the FRET rate constant based on the orientation factor. In Ref.^27^, the DD approximation was numerically assessed by comparing its predictions of electronic coupling on MD snapshots of poly-l-proline oligomers with those simulated by the transition density cube (TDC) method as reliable reference^33^. The TDC method allows for accurate determination of electronic coupling at all interchromophore DA distances. Reference^27^ showed an intriguing observation that the Förster theory can break down at short interchromophore distances because its DD approximation causes marked errors in the predictions of electronic coupling at these distances.

In this study, we investigate the value of the orientation factor towards the reliable FRET analysis. It can be computationally determined on top of the accurate prediction of the electronic coupling between a DA pair of fluorophores. Furthermore, the statistical average to account for the anisotropic nature of the movement of DA molecules needs to be considered, thus requiring extensive electronic coupling calculations on a considerable number of MD snapshot structures. Despite its high accuracy, a major disadvantage of the TDC method lies in its high computational expense, which is ascribed to the complexity to treat the interactions between the transition densities in a quantum mechanical manner. The transition charge from electrostatic potential (TrESP)^34–36^ method is a promising alternative, which is based on classical Coulomb interactions using atomic transition charges, allowing for efficient electronic coupling calculations. The accuracy of TrESP^35^ was investigated by one of the authors (K.J.F.) comparing the electronic couplings calculated by the TrESP method with those by the transition-density-fragment interaction (TDFI)^35,37–39^ method. Note that the TDFI method is even more accurate than the TDC method in terms of the treatment of the two electron integrals and transition densities^35^. The results showed that despite its simplicity, the TrESP offered surprisingly accurate predictions with an error of within 5 cm^−1^ compared to the TDFI method with the two chromophores separated by over 4 Å^35^. This indicates that the usage of the TrESP method is appropriate for most FRET systems. In fact, the validity of the TrESP method for FRET systems was supported by Sobakinskaya et al.^32^ They performed MD simulations and TrESP calculations on polyproline helices labeled with two chromophores and showed that this approach is very effective for quantitative interpretation of FRET experiments.

Despite the inappropriate assumptions in the Förster theory as mentioned above, its simplicity remains attractive and thus keeps its use for FRET analysis still widely practiced. In this study, intimately related to the earlier works^23,27,32^, our focus is particularly on the inappropriateness in the isotropic treatment with $\kappa^{2}$ = 2/3 for inferring the interchromophore distance. We have examined the previous experimental FRET study on a biosystem by Yoneda et al.^40–42^ as an interesting case. References^40–42^ performed the FRET analysis to rationalize the location of the artificial chromophore (Alexa Fluor 647: A647) covalently attached to light-harvesting 2 complex (LH2) from purple photosynthetic bacteria^43–46^. The analysis was based on the Förster theory including *κ*^2^ = 2/3 in conjunction with the FRET rate constants measured by using the ultrafast transient absorption spectroscopy.

The LH2 used in this study is a pigment-protein complex in a purple photosynthetic bacterium *Rhodoblastus acidophilus* strain 10050^43–46^. It contains 27 bacteriochlorophyll *a* (BChl), which form two types of ring-shaped BChl aggregates with different radii. The inner and outer rings, consisting of 18 and 9 BChls, respectively, are named B850 and B800, respectively, due to their absorption wavelengths^47^. The formation of such BChl aggregates leads to efficient light harvesting^48–50^. The light energy absorbed by B800 is transferred to B850 with a time constant of approximately 700 fs^51^. The collected excitation energy is further transferred to the core antenna–reaction center complex (LH1-RC) and utilized for photosynthetic reactions^43,52,53^. Yoneda et al. successfully demonstrated the improvement of light absorption efficiency by the conjugation of A647 with LH2, which is attributed to the efficient EET from A647 to BChl^40^. They then surmised the possible location of A647 in the LH2-A647 using the measured data of FRET rate constants on the basis of the Förster equation^40–42^. While they have made great achievements in artificially modifying protein functions, the prediction of the location of A647 remains to be reviewed because they used an orientation factor of 2/3 for the intermolecular distance calculation as mentioned earlier. Although the LH2-A647 exhibited no signals of A647 in the linear dichroism spectrum indicative of an averaged orientation, A647 is covalently bound to LH2 via a linker in a crowded environment, which may restrict the molecular orientation. Accurate prediction of A647 position in LH2 is expected to lead to further improvement of FRET efficiency by modifying the linker.

To address the questions and concerns in the theoretical treatment of the FRET analysis shown by Yoneda et al., this study has investigated the magnitude of the orientation factor between A647 and B850-BChl by the computational means that can go beyond Förster theory using MD simulations and electronic coupling calculations by the TrESP method. We have examined how much the orientation factor determined by the MD-based approach would alter the prediction of A647 position compared to the isotropic mean value, given the experimental FRET rate constant. Furthermore, we have analyzed the orientation of A647 using Euler angles, which provides insight into enhancing the FRET efficiency. The present approach would be useful for further extending the applicability of FRET analysis.

## Methods

### Intermolecular distance calculation using FRET rate constant

In this section, we provide a theoretical overview of how to determine the intermolecular distance from the given FRET rate constant. For more details, please refer to Refs.^2–4^ and others.

The FRET rate constant $k_{{\text{T}}}$ is represented by

1 $$k_{{\text{T}}} = \frac{{9\left( {\ln 10} \right)\kappa^{2} \varphi_{{\text{D}}} }}{{128\pi^{5} N_{{\text{A}}} n^{4} \tau_{{\text{D}}} R_{{{\text{DA}}}}^{6} }}J.$$

Other than the Avogadro constant $N_{{\text{A}}}$, Eq. (1) hinges on six parametric variables: (i) $\kappa^{2}$ is the orientation factor between the transition dipoles of the donor (D) and acceptor (A); (ii) $R_{{{\text{DA}}}}$ is the distance between the donor and acceptor; (iii) $J$ is the overlap between the fluorescence spectrum of the donor and the absorption spectrum of the acceptor; (iv) $n$ is the refractive index; (v) $\varphi_{{\text{D}}}$ is the fluorescence quantum yield of the donor; (vi) $\tau_{{\text{D}}}$ is the fluorescence lifetime of the donor in the absence of the acceptor. It should be noted that although $\kappa$ is sometimes referred to as the orientation factor, this study uses the square of $\kappa$ as the orientation factor. Removing $k_{{\text{T}}}$ and $\tau_{{\text{D}}}$ in Eq. (1) by setting $k_{{\text{T}}}$ to the inverse of $\tau_{{\text{D}}}$, we can define the so-called Förster distance (or Förster radius) denoted $R_{0}$ hereafter as follows:

2 $$R_{0} = \left( {\frac{{9\left( {\ln 10} \right)\kappa^{2} \varphi_{{\text{D}}} }}{{128\pi^{5} N_{{\text{A}}} n^{4} }}J} \right)^{\frac{1}{6}} ,$$

which is given as a function of four parameters: $\kappa^{2}$, $J$, $n$, and $\varphi_{{\text{D}}}$. Using Eqs. (1) and (2), the FRET efficiency $\eta_{{\text{F}}}$ can be written as

3 $$\eta_{{\text{F}}} = \frac{{k_{{\text{T}}} }}{{k_{{\text{T}}} + \tau_{{\text{D}}}^{ - 1} }} = \left[ {1 + \left( {\frac{{R_{{{\text{DA}}}} }}{{R_{0} }}} \right)^{6} } \right]^{ - 1} .$$

As can be confirmed from Eq. (3), $\eta_{{\text{F}}}$ is 0.5 when $R_{{{\text{DA}}}}$ is equal to $R_{0}$. This means that the Förster distance $R_{0}$ corresponds to the intermolecular distance with a FRET efficiency of 50%.

Substituting Eq. (2) into Eq. (1) gives

4 $$R_{{{\text{DA}}}} = R_{0} \left( {k_{{\text{T}}} \tau_{{\text{D}}} } \right)^{{ - \frac{1}{6}}} .$$

Equation (4) is often used to determine $R_{{{\text{DA}}}}$ from the measured $k_{{\text{T}}}$ in FRET analysis. In this study, it indeed plays a central role in calculating the distance between A647 and BChl in combination with the experimental data.

### Determination of orientation factor using TrESP method

We now turn to the orientation factor $\kappa^{2}$ in Eq. (1). As will be detailed later, it originates with the DD approximation to the electronic coupling between donor and acceptor. Let us here briefly overview the electronic coupling, denoted $V$. It is an intermediate physical quantity that describes the interaction between different electronic states and is expressed by^38,39,54^

5 $$\begin{aligned} V = & \int {{\text{d}}{\mathbf{r}}} \int {{\text{d}}{\mathbf{r}}^{'} } \frac{{\rho _{{\text{D}}}^{{t^{*} }} \left( {{\mathbf{r}},{\mathbf{r}}} \right)\rho _{{\text{A}}}^{t} \left( {{\mathbf{r}}^{'} ,{\mathbf{r}}^{'} } \right)}}{{4\pi \varepsilon _{0} n^{2} \left| {{\mathbf{r}} - {\mathbf{r}}^{'} } \right|}} - \frac{1}{2}\int {{\text{d}}{\mathbf{r}}} \int {{\text{d}}{\mathbf{r}}^{'} } \frac{{\rho _{{\text{D}}}^{{t^{*} }} \left( {{\mathbf{r}},{\mathbf{r}}^{'} } \right)\rho _{{\text{A}}}^{t} \left( {{\mathbf{r}}^{'} ,{\mathbf{r}}} \right)}}{{4\pi \varepsilon _{0} n^{2} \left| {{\mathbf{r}} - {\mathbf{r}}^{'} } \right|}} \\ \equiv & V_{{{\text{Coul}}}} + V_{{{\text{Exch}}}} , \\ \end{aligned}$$

where $\rho_{X}^{t} ({\mathbf{r}},{\mathbf{r}})$ is the one-electron transition density of molecule *X* (*X* *=* D or A), ${\mathbf{r}}$ is the spatial coordinate of the electron, and $\varepsilon_{0}$ is the vacuum permittivity. The first and second terms correspond to the Coulomb $V_{{{\text{Coul}}}}$ and exchange $V_{{{\text{Exch}}}}$ interactions, respectively. The contribution of the $V_{{{\text{Exch}}}}$ interaction, related to the so-called Dexter mechanism^55^, is considered to be large only when the intermolecular distance is very small, and thus can be neglected in most FRET systems. Interestingly, previous studies showed that the contribution of $V_{{{\text{Exch}}}}$ is somewhat small even when the intermolecular distance is very small (~ 3.5 Å) in ethylene dimers, but the contribution of EET via the charge transfer states is large instead^38,56^.

The DD approximation, which is derived from the leading term of the multipole expansion of $V_{{{\text{Coul}}}}$, gives^1^

6 $$\begin{aligned} V_{{{\text{Coul}}}}^{{{\text{DD}}}} &= \frac{{{{\varvec{\upmu}}}_{{\text{D}}} \cdot {{\varvec{\upmu}}}_{{\text{A}}} - 3\left( {{{\varvec{\upmu}}}_{{\text{D}}} \cdot {\mathbf{e}}} \right)\left( {{{\varvec{\upmu}}}_{{\text{A}}} \cdot {\mathbf{e}}} \right)}}{{4\pi \varepsilon_{0} n^{2} R_{{{\text{DA}}}}^{3} }} \\ &= \frac{{\left| {{{\varvec{\upmu}}}_{{\text{D}}} } \right|\left| {{{\varvec{\upmu}}}_{{\text{A}}} } \right|\kappa }}{{4\pi \varepsilon_{0} n^{2} R_{{{\text{DA}}}}^{3} }}, \\ \end{aligned}$$

with

7 $$\kappa = \frac{{{{\varvec{\upmu}}}_{{\text{D}}} \cdot {{\varvec{\upmu}}}_{{\text{A}}} - 3\left( {{{\varvec{\upmu}}}_{{\text{D}}} \cdot {\mathbf{e}}} \right)\left( {{{\varvec{\upmu}}}_{{\text{A}}} \cdot {\mathbf{e}}} \right)}}{{\left| {{{\varvec{\upmu}}}_{{\text{D}}} } \right|\left| {{{\varvec{\upmu}}}_{{\text{A}}} } \right|}}.$$

Here, ${{\varvec{\upmu}}}_{{\text{D}}}$ and ${{\varvec{\upmu}}}_{{\text{A}}}$ are the transition dipoles of the donor and acceptor, respectively, and ${\mathbf{e}}$ is the unit vector connecting between ${{\varvec{\upmu}}}_{{\text{D}}}$ and ${{\varvec{\upmu}}}_{{\text{A}}}$. The orientation factor $\kappa^{2}$ can be obtained from the dot products in Eq. (7) as follows:

8 $$\kappa^{2} = \left( {\cos \theta_{{{\text{DA}}}} - 3\cos \theta_{{\text{D}}} \cos \theta_{{\text{A}}} } \right)^{2} ,$$

where $\theta_{{{\text{DA}}}}$, $\theta_{{\text{D}}}$, and $\theta_{{\text{A}}}$ denote the angles between ${{\varvec{\upmu}}}_{{\text{D}}}$ and ${{\varvec{\upmu}}}_{{\text{A}}}$, ${{\varvec{\upmu}}}_{{\text{D}}}$ and ${\mathbf{e}}$, and ${{\varvec{\upmu}}}_{{\text{A}}}$ and ${\mathbf{e}}$, respectively. It should be noted that Eq. (8) describes the square of $\kappa$ as the orientation factor; $\kappa^{2}$ arises because the square of the interaction potential is used in calculating the square of the matrix element governing the transition rate according to Fermi’s golden rule.

The DD method has the advantage of simplifying the electronic coupling calculations in Eq. (5) and providing an intuitive understanding of electronic coupling. However, there is a problem that the applicability of the DD method is limited to the case where the intermolecular distance is larger than the molecular sizes. To avoid such a problem in the DD method, we use the TrESP method^34–36^, in which the electronic Coulomb coupling $V_{{{\text{Coul}}}}$ is represented by classical Coulomb interactions between transition charges.

9 $$V_{{{\text{Coul}}}}^{{{\text{TrESP}}}} = \sum\limits_{{i \in {\text{D}}}} {\sum\limits_{{j \in {\text{A}}}} {\frac{{q_{i} q_{j} }}{{4\pi \varepsilon_{0} n^{2} r_{ij} }}} } ,$$

where $q_{i}$ is the transition charge on atom *i* and $r_{ij}$ is the interatomic distance. In contrast to the DD method, which calculates the electronic couplings based on the transition dipoles each placed at the molecular center (e.g., the center of mass), the TrESP method describes the interactions of the transition charges assigned to each atom in a molecule. By handling such multi-centric interactions, the TrESP method enables more accurate electronic coupling calculations than the DD method. In other words, the TrESP method incorporates interactions between multipoles of higher order than dipoles, which the DD method does not include. In addition, the TrESP method is computationally inexpensive due to its classical description, which makes it applicable to systems containing many chromophores^57^.

The orientation factor $\kappa^{2}$ again essentially rises from the low-order (or DD) approximation (Eq. (6)), and the TrESP coupling (Eq. (9)) is formally $\kappa^{2}$-free. Even though the TrESP method is superior in terms of accuracy, the characterization based on $\kappa^{2}$ is yet useful to comprehend a spatial relation of the electronic coupling of the DA pair. To this end, we here introduce an effective orientation factor $\kappa_{{{\text{eff}}}}^{2}$ that reflects the TrESP based electronic coupling. It is determined such that a single DD interaction of the DA molecules with this $\kappa_{{{\text{eff}}}}^{2}$ exactly yields the TrESP coupling. This can thus be derived by equating Eq. (6) to Eq. (9), resulting in the following formula:

10 $$\kappa_{{{\text{eff}}}}^{2} = \left( {\frac{{R_{{{\text{DA}}}}^{3} }}{{\left| {{{\varvec{\upmu}}}_{{\text{D}}} } \right|\left| {{{\varvec{\upmu}}}_{{\text{A}}} } \right|}}\sum\limits_{{i \in {\text{D}}}} {\sum\limits_{{j \in {\text{A}}}} {\frac{{q_{i} q_{j} }}{{r_{ij} }}} } } \right)^{2} .$$

This equation is built upon the electronic coupling determined by the TrESP method, allowing ones to account for the effect of dipole–dipole and higher-order multipole interactions. This equation is used throughout this study to evaluate the magnitude of the orientation factor, unless otherwise noted. The interaction nature may be interpreted by replacing it with a simpler form based on the interaction of a pair of virtual (or effective) dipoles.

### Computational details

The atomic coordinates of LH2 for the crystal structure were taken from protein data bank (PDB) entry 1NKZ^46^. A647 was attached to Lys51 located at the C-terminal side of the LH2α polypeptide chain by uisng a linker reagent (*N*-{6-[3-(2-pyridyldithio)propionamido]hexanoyl}sulfosuccinimide)^40,42^, as in the experimental conditions^42^. The chemical structures of the linkage and A647 were shown in Refs.^40,42^. Under the reaction conditions, A647s are attached to Lys51 and Lys5. The previous studies indicated that the A647 attached to Lys51 exclusively acts as the energy donor to B850 in the lipid bilayer environment while the energy transfer activity of the A647 attached to Lys5 is negligible^42^. Therefore, we constructed the calculation model composed of LH2 and Lys51-attached A647 in this study. MD simulations were performed on the LH2-A647 conjugate and a 1-palmitoyl-2-oleoyl-phosphatidylethanol-amine (POPE) membrane modeled in a periodic boundary box (134 × 133 × 129 Å^3^) using a time step of 2 fs under NPT conditions at 300 K and 1 atm. In the 100 ns MD simulation for equilibration, the fluctuations of root mean square deviation (RMSD) were nearly constant after 80 ns, followed by an additional 30 ns MD simulation (total of 130 ns). The particle mesh Ewald (PME) method^58^ was applied to nonbonding interactions and the SHAKE method^59^ was used for distance constraint of the bonds including hydrogens. The temperature was gradually raised from 0 K to 300 K and maintained using a Langevin thermostat^60^. To keep the charge state of the entire system neutral, 13 sodium ions were added. A total of 208,609 atoms were included in the periodic boundary box. The TIP3P^61^, ff14SB^62^, and lipid14^63^ force fields were used for water molecules, the protein, and POPE, respectively. The general Amber force field (GAFF)^64^ was used for BChl, A647, and rhodopin β-d-glucoside.

In the excited state calculations, time-dependent density-functional theory (TDDFT) with the CAM-B3LYP functional^65^ (TD-CAM-B3LYP) was employed for obtaining the transition densities and transition dipoles. The 6-31G(d) basis set was used for atomic basis functions. Our focus was on FRET from the first excited state of A647 to the first excited state of B850-BChl. In both A647 and B850-BChl, the first excited state was characterized as π–π^*^ excitation with a large oscillator strength. No degeneracy was observed in either excited state. Therefore, the transition densities of these states were used to determine the transition charges by the ESP fitting procedure^66^. The magnitude of the vacuum transition dipole moment of BChl has been experimentally measured to be 6.09 Debye^67^, whereas the transition dipole moment of the first excited state at the TD-CAM-B3LYP/6-31G(d) level was calculated to be 7.50 Debye. This indicates that the present calculation overestimates the experimental value. However, the magnitude of the transition dipole moment has no effect on the orientation factor because it is formally cancelled during the calculation. Therefore, the transition dipole (and transition charge) values calculated without scaling were used in this study.

**Figure 1:**
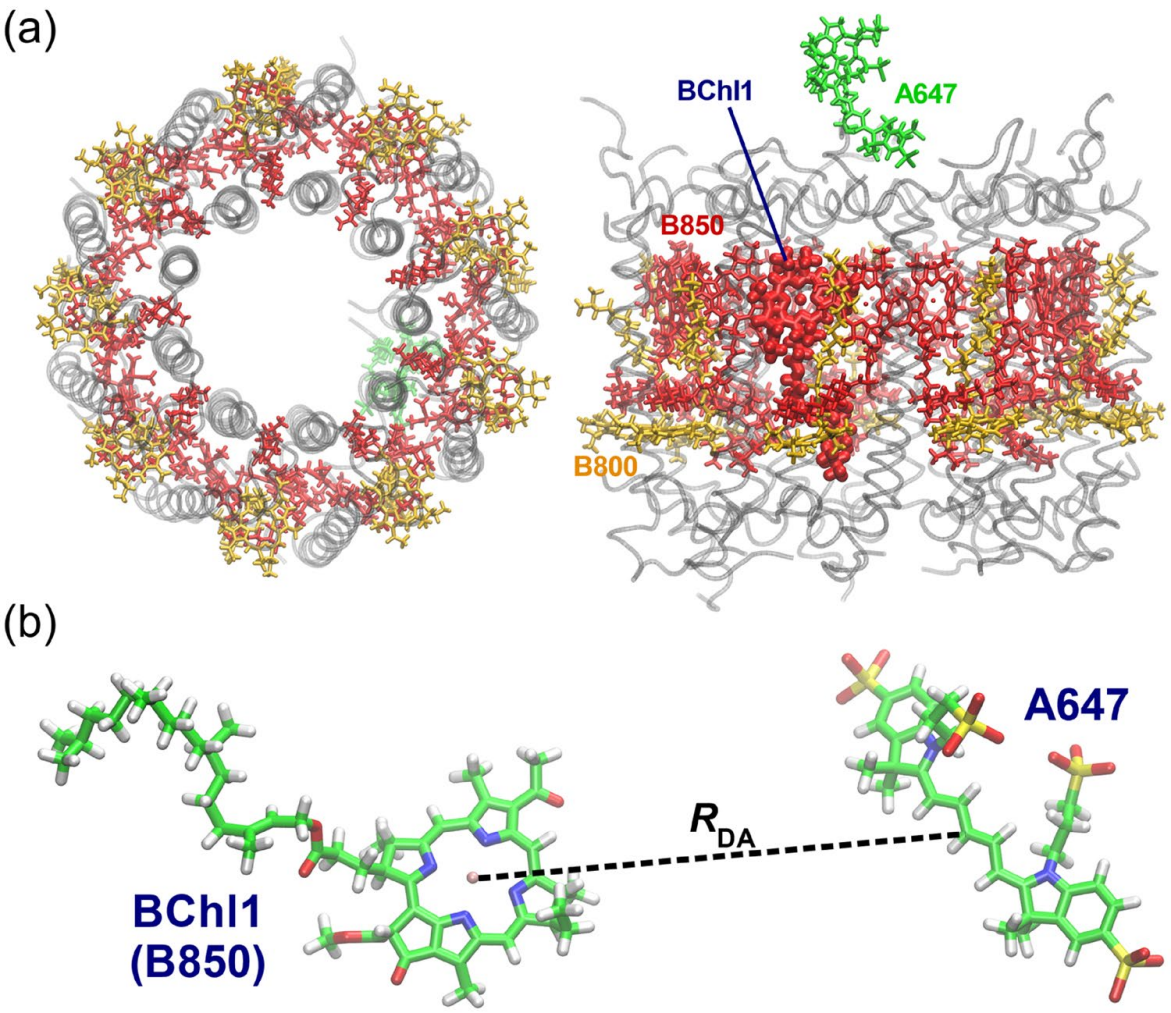
(**a**) Structure of LH2-A647 conjugate after 100 ns MD simulation. The top view is shown on the left and the side view on the right. The structures of 18 BChls in B850, 9 BChls in B800, and A647 are shown in red, orange, and green, respectively. The structure of BChl1, the closest BChl to A647, is represented by the thick licorice. (**b**) Definition of the intermolecular distance between A647 and BChl1. The distance between the center of mass of the π-conjugated plane of A647 and the Mg atom of BChl1 is defined as $R_{{{\text{DA}}}}$.

From the geometric data of LH2 offered by the MD simulation, we can directly measure the DA intermolecular distance $R_{{{\text{DA}}}}$. Such MD geometry-based $R_{{{\text{DA}}}}$ is hereafter denoted $R_{{{\text{DA}}}}^{{{\text{MD}}}}$. In this study, it was calculated as the distance between the center of mass of the π-conjugated plane of A647 and the Mg atom of BChl1 at the snapshot geometry of the MD trajectory, as shown in Fig. 1b.

With such obtained $R_{{{\text{DA}}}}^{{{\text{MD}}}}$, the effective orientation factor $\kappa_{{{\text{eff}}}}^{2}$ based on the TrESP method was determined using Eq. (10) with $q_{i}$ set to the transition charges resulting from the aforementioned TD-CAM-B3LYP calculations along with the computed real transition dipoles for ${{\varvec{\upmu}}}_{{\text{D}}}$ and ${{\varvec{\upmu}}}_{{\text{A}}}$. In addition, the calculation of $\kappa^{2}$ at the level of the DD approximation was carried out where necessary, using Eq. (8) with the TD-CAM-B3LYP-level ${{\varvec{\upmu}}}_{{\text{D}}}$ and ${{\varvec{\upmu}}}_{{\text{A}}}$.

Given the orientation factor $\kappa^{2}$ determined by fully computational approaches, the Förster theory-based estimation of the intermolecular distance $R_{{{\text{DA}}}}$ was done using Eq. (4). The prerequisite parameters in Eq. (4) other than $\kappa^{2}$ were taken from the experimental work of Yoneda et al:^42^ a fluorescence quantum yield of 0.2 for $\varphi_{{\text{D}}}$, a fluorescence lifetime of 0.8 ns for $\tau_{{\text{D}}}$, a FRET rate constant of 2.7 × 10^11^ s^−1^ for $k_{{\text{T}}}$, a spectral overlap of 2.6 × 10^−13^ cm^6^ for *J,* and a refractive index of 1.45 for *n*. Again note that $\kappa^{2}$ is conventionally set to a constant 2/3 representing the isotropic mean value.

The Gaussian16 program package^68^ was used to calculate the excited states of BChl and A647. All MD simulations were performed with the AMBER 2019 program package^69^.

## Results and discussion

As discussed earlier, we can determine the orientation factor $\kappa^{2}$ in a fully computational manner based on MD simulation and TDDFT excited-state calculation. This capability of the modern computational approaches is extremely powerful to elucidate the molecular-level details. In this section, the scheme to predict $\kappa^{2}$ and related $R_{{{\text{DA}}}}$ is examined by applying it to the LH2-A647 conjugate as a real test case, which is based on the previous experimental works^42^. Our calculation of the orientation factor $\kappa^{2}$ (Eq. (10)) can be explicitly linked to the electronic coupling at the level of the TrESP theory, as written in “Methods” section. In this section, we first discuss the prediction of $\kappa^{2}$ and $R_{{{\text{DA}}}}$ for a single MD snapshot in comparison with the variant with the DD-approximated electronic coupling. The statistically-averaged $\kappa^{2}$ and $R_{{{\text{DA}}}}$ accounting for the structural fluctuation in the MD simulation are next shown, compared with the isotropic mean values. Then, these results are further analyzed compared with the experimental conditions. Finally, a theoretical attempt to enhance the FRET efficiency in the LH2-A647 conjugate by modulating the A647’s orientation is shown and discussed.

### Comparison of electronic coupling values obtained with DD and TrESP

We first analyzed the orientation factor $\kappa^{2}$ and $R_{{{\text{DA}}}}^{{{\text{MD}}}}$ using the structure obtained from the 100 ns MD simulation. Figure 1a shows the MD snapshot at 100 ns, where A647 is located closest to BChl1 of B850, and its intermolecular distance is 25.3 Å. Electronic couplings between 18 BChls and A647 in B850 were calculated using the TrESP method, yielding values ranging from 0.17 to 55.7 cm^−1^. The largest electronic coupling (55.7 cm^−1^) was obtained from BChl1. The second and third largest electronic couplings (40.6 and 20.9 cm^−1^) are located on the two sides of BChl1, with their intermolecular distances of 26.3 and 27.8 Å, respectively. Based on these results, we proceeded to analyze BChl1, which has the largest electronic coupling. As summarized in Table 1, the DD method gave the electronic coupling of 61.1 cm^−1^ for BChl1. The electronic couplings by the TrESP and DD methods were used to determine the orientation factors $\kappa^{2}$, which gave values of 1.63 and 1.96, respectively. These results indicated that the DD method gives a larger electronic coupling than the TrESP method, as consistent with the previous reports^27,35^.

**Table 1:** Electronic coupling energies in absolute value (|*V*_Coul_|) and orientation factors ($\kappa^{2}$) obtained with the TrESP (Eq. (10)) and DD methods (Eq. (8)). Isotropic mean of $\kappa^{2}$ is included for comparison.

	|*V*_Coul_| (cm^−1^)	*κ*^2^
TrESP	55.7	1.63 (1.55)^a^
DD	61.1	1.96
Isotropic mean	–	0.67^b^

^a^Value in parenthesis is the average of the orientation factors shown in Fig. 3.

^b^Corresponding to 2/3.

**Figure 2:**
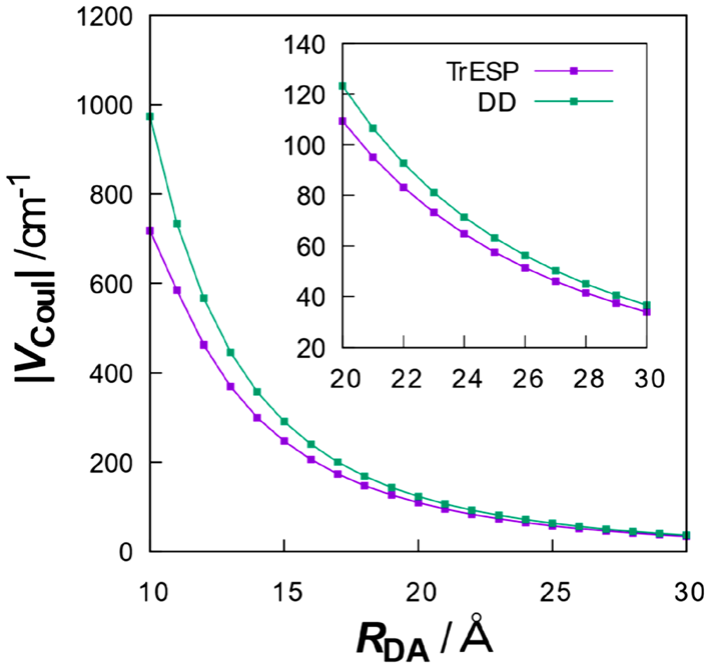
Electronic coupling values as a function of intermolecular A647-BChl1 distance $R_{{{\text{DA}}}}$. The definition of $R_{{{\text{DA}}}}$ is shown in Fig. 1b.

To further examine the difference between the TrESP and DD methods, we calculated the electronic coupling values as a function of the varying intermolecular distance for these structures with the other parameters including real transition dipoles and densities unchanged. We again considered the intermolecular distance $R_{{{\text{DA}}}}$ defined in Fig. 1b. As can be seen in Fig. 2, the absolute value of the electronic coupling increased with decreasing intermolecular distance. It is also shown that the DD method gives larger electronic couplings than the TrESP method for the small intermolecular distance. This is due to the fact that the DD method is an approximation that is valid only when the intermolecular distance is larger than their molecule sizes. It was also found that an intermolecular distance larger than 26.0 Å is required for the DD method to satisfy the accuracy within an error of 5.00 cm^−1^, comparable to the results of the TrESP method. As mentioned above, the MD structure had an intermolecular distance of 25.3 Å between A647 and BChl1 for $R_{{{\text{DA}}}}^{{{\text{MD}}}}$. These results confirmed that the DD method may not be suitable for the analysis of electronic coupling in the LH2-A647 conjugate in some cases. In the following analysis, the electronic coupling values calculated with the TrESP method were used to gauge the orientation factor.

### Analysis of the orientation factor between A647 and BChl

**Figure 3:**
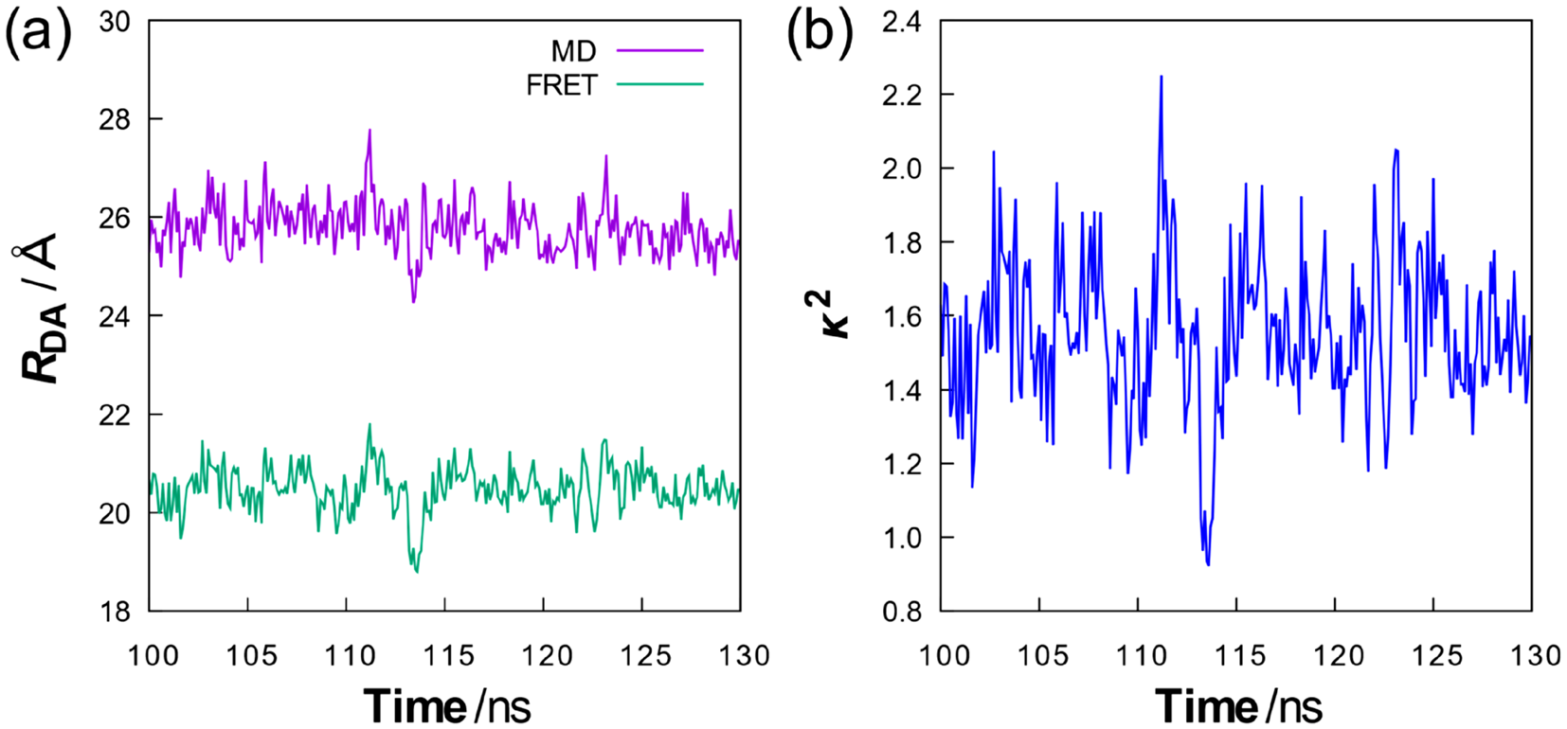
Intermolecular distances and orientation factors as a function of time. (**a**) A647-BChl1 distances $R_{{{\text{DA}}}}$ obtained from MD simulation and Eq. (4), (**b**) orientation factors $\kappa^{2}$ obtained from electronic coupling calculations with the TrESP method (Eq. (10)).

We next analyzed the time evolution of the intermolecular distance between A647 and BChl1 and the values of the orientation factor. For this purpose, an additional 30 ns MD simulation was performed on the structure obtained from the 100 ns simulation, and the electronic couplings were calculated for the resulting MD structures in 0.1 ns increments using the TrESP method. As shown in Fig. 3a, the intermolecular distance between A647 and BChl1 was 24.3–27.8 Å in the time region of 100–130 ns, indicating that A647 does not move significantly as the system equilibrates. This is probably due to the strong interaction of A647 with the protein as it is located near the interface between the protein and water. In this time region, the orientation factor of A647-BChl1 was determined to be 0.922–2.25 (Fig. 3b), which is larger than the isotropic mean value 2/3. By substituting these values of the orientation factor into Eq. (4), we further estimated the intermolecular distance of A647-BChl1 at each time. Such distance calculations are useful for evaluating the validity of the determined orientation factors. As also shown in Fig. 3a, the intermolecular distance was calculated to be 18.8–21.8 Å, which was closer to the MD result (24.3–27.8 Å) than the value obtained for $\kappa^{2}$ = 2/3 (17.8 Å). This result also showed that the time evolution of the intermolecular distances based on Eq. (4) is relatively similar to that of the MD results, confirming the validity of the present approach for this system.

We further investigated the average value of the orientation factor of A647-BChl1 in the time region of 100–130 ns. As shown in Table 1, the average value of the orientation factor at each time was calculated to be 1.55, which deviated significantly from the isotropic mean value (2/3). This was attributed to the fact that the movement of A647 was greatly restricted by the linker. Substituting the orientation factor of $\kappa^{2}$ = 1.55 into Eq. (4) yielded an intermolecular distance of 20.5 Å, indicating that $\kappa^{2}$ = 1.55 reproduces the mean value of the MD results (25.8 Å) in better agreement than $\kappa^{2}$ = 2/3 (17.8 Å). We also averaged the intermolecular distances derived by substituting the orientation factors at each time into Eq. (4) (Fig. 3a). The resulting distance was 20.5 Å, which was consistent with the intermolecular distance obtained from the average value of the orientation factor ($\kappa^{2}$ = 1.55). This result confirmed that the averaged orientation factor determined by this approach is useful for calculating the A647-BChl1 distance.

### Analysis of spectral overlap and refractive index

The above analysis successfully demonstrated that the average value of the orientation factor determined in this study, $\kappa^{2}$ = 1.55, is more suitable for calculating the intermolecular distance than the isotropic mean value, 2/3. Nonetheless, this refined intermolecular distance ($R_{{{\text{DA}}}}$) did not fully reproduce the MD result ($R_{{{\text{DA}}}}^{{{\text{MD}}}}$), still underestimated by 5.3 Å. This error seemingly stems from the fact that the relation between $\kappa^{2}$ and *R*_DA_ via Eq. (4) further hinges on various other physical parameters, such as $J$, $n$, $\varphi_{{\text{D}}}$, and $\tau_{{\text{D}}}$ (see Eq. (1)). As mentioned earlier, we re-used the values of these parameters that were previously used in the modeling of Yoneda et al.^42^ for their estimation of $R_{{{\text{DA}}}}$. Here we touch on two parameters separately: the spectral overlap *J* and the refractive index *n*.

First, let us remark on the value of *J*. In the previous section, we used a spectral overlap of 2.6 × 10^−13^ cm^6^, which was taken from the experimental measurement in a micellar solution^40–42^. However, this value may be different from the actual value because A647 is bound to the LH2. To examine to what degree the value of $J$ affects the inference of $R_{{{\text{DA}}}}$, we calculated the intermolecular distance as a function of varying $J$. Table 2 shows that the $R_{{{\text{DA}}}}$ can be predicted to be increasingly larger with increasing $J$; however, the calculated $R_{{{\text{DA}}}}$ was found to be quite robust with an increase in $J$ relative to that of the micellar solution. This reflects the fact that $R_{{{\text{DA}}}}$ is proportional to the one-sixth power of $J$ according to Eqs. (2) and (4). If we solely reparameterize the value of $J$ so as for the predicted $R_{{{\text{DA}}}}$ to approach $R_{{{\text{DA}}}}^{{{\text{MD}}}}$, it has to be as much as four times larger than $J$ of the micellar solution. Of course, this would be unrealistic. Therefore, even if actual $J$ in the membrane-embedded LH2 is available, its use should not make an appreciable difference in the inference of $R_{{{\text{DA}}}}$ compared to $J$ in the micellar solution. Still, larger $J$ has a certain but minor impact on enlarging $R_{{{\text{DA}}}}$.

**Table 2:** Effect of spectral overlap (*J*) and refractive index (*n*) on intermolecular distance (*R*_DA_).

*κ*^2^	*J* (×10^−13^ cm^6^)	*n*	*R*_DA_^a^ (Å)	$R_{{{\text{DA}}}}^{{{\text{MD}}}}$^b^ (Å)
1.55	2.00	1.45	19.6	25.8
	2.60		20.5	
	3.00		21.0	
	4.00		22.0	
	5.00		22.9	
	10.3		25.8	
	2.60	1.00	26.3	
		1.03	25.8	
		1.10	24.7	
		1.20	23.3	
		1.45	20.5	
		1.60	19.2	

^a^Intermolecular distance determined by Eq. (4).

^b^Average of intermolecular distances obtained from a 100–130 ns MD simulation.

Second, we turn to the value of the refractive index $n$. In the previous section, a refractive index of $n$ = 1.45 was adopted, which corresponds to a uniform membrane environment^70^. However, the result of the MD simulation showed that A647 is in a mixed environment of water and membrane, indicating that the value of $n$ = 1.45 does not wholly reflect the simulated system. Equations (2) and (4) indicate that $R_{{{\text{DA}}}}$ is inversely proportional to the four-sixths power of $n$*.* This means that $n$ has a more pronounced effect on $R_{{{\text{DA}}}}$ than $J$. The $n$-dependence of the prediction of $R_{{{\text{DA}}}}$ was simulated (Table 2). As summarized, when the refractive index $n$ is set to a value smaller than 1.45, the error of $R_{{{\text{DA}}}}$ is reduced. Note that the error can vanish when $n$ = 1.03, which nearly corresponds to a refractive index of vacuum; thus, the single-parameter refitting again overcorrects the model. Overall, the trend of the calculated $R_{{{\text{DA}}}}$ as a function of $n$ exhibits a well-suited feature because a non-uniform environment of water ($n$ = 1.33) and membrane in our simulation should have a smaller $n$ than the uniform membrane environment. If $n$ is smaller, then it causes a weaker screening effect on electronic coupling. The magnitude of the refractive index cannot be further discussed in this analysis because strict quantitativeness of the other parameters including $\kappa^{2}$ also plays a role.

**Figure 4:**
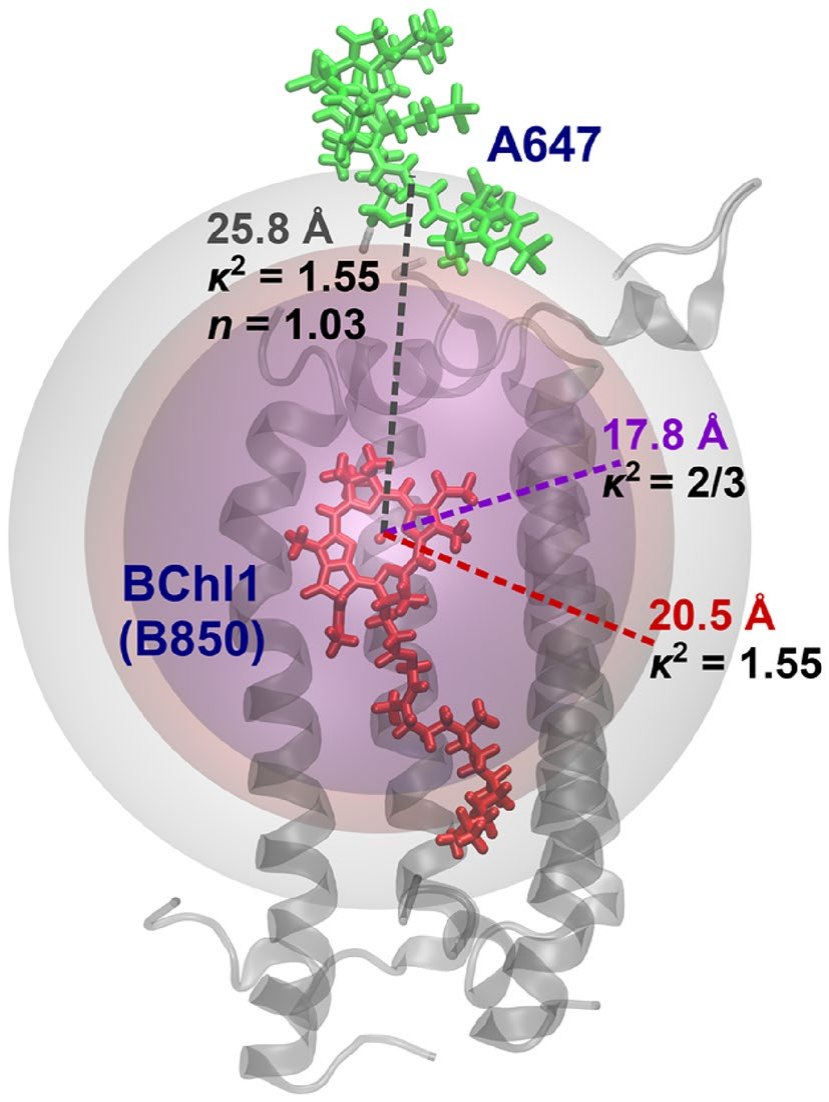
Sphere representing the A647 position predicted by FRET analysis. The orientation factor $\kappa^{2}$ = 1.55 obtained from the electronic coupling calculation gives a better prediction of the A647 position than the isotropic mean value of 2/3. Further consideration of the refractive index *n* to 1.03 leads to a near perfect match with the A647 position in the MD result.

In order to clearly compare the calculated intermolecular distances, the predicted position of A647 is represented by a sphere around the center of mass of BChl1 in Fig. 4. Here, we consider the closeness of the sphere to the A647 position in the MD structure as the accuracy of the prediction. We can see that the sphere for $\kappa^{2}$ = 1.55 is located closer to A647 than that for $\kappa^{2}$ = 2/3. Furthermore, the sphere for $\kappa^{2}$ = 1.55 and *n* = 1.03 is in contact with the A647 position. These results again indicate that in addition to the orientation factor, the value of the other parameters such as the refractive index should also be properly examined for an accurate estimation of the intermolecular distance.

### Exploration of A647 orientations using Euler angles

**Figure 5:**
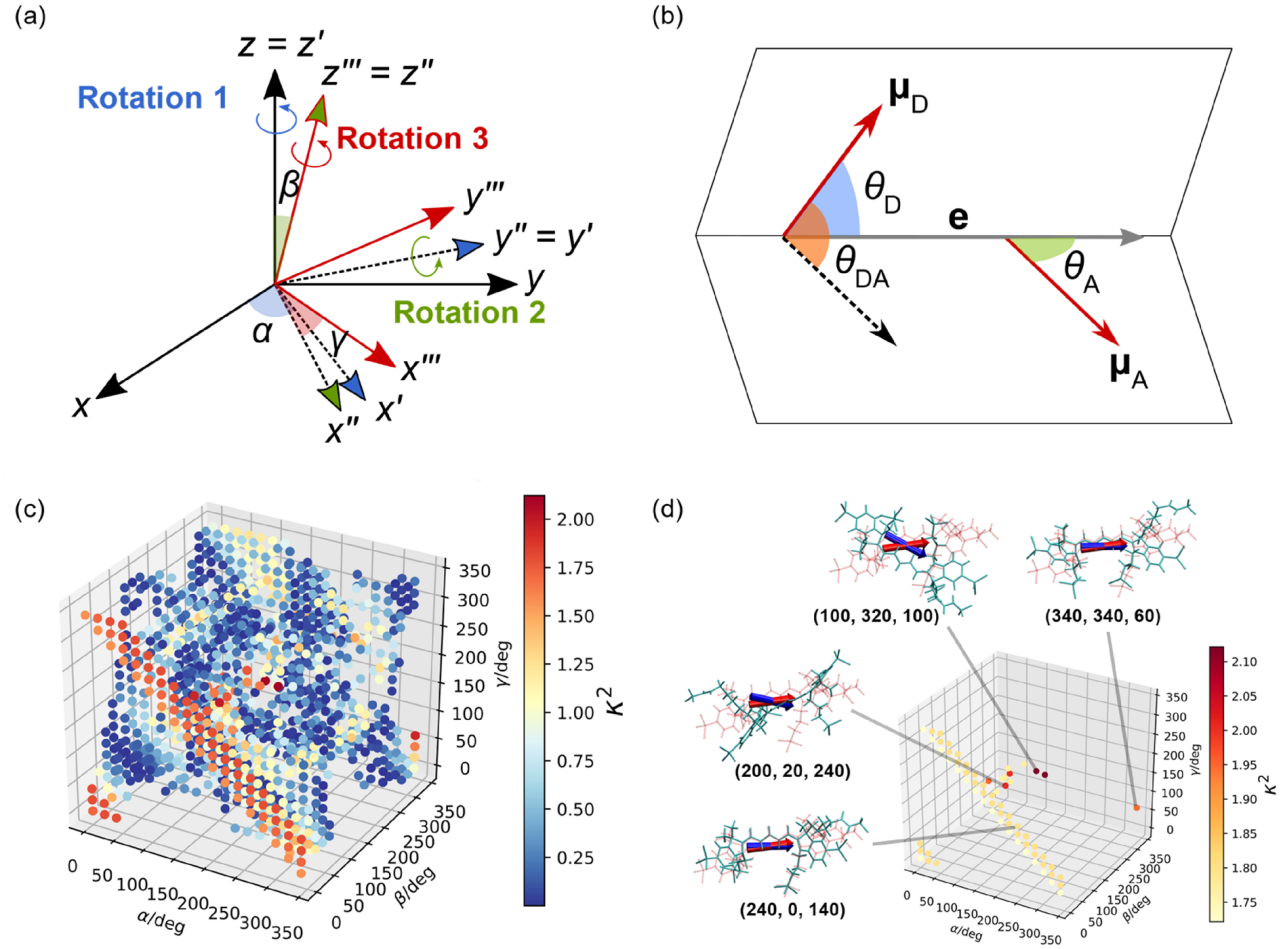
(**a**) Schematic illustration of Euler angles, (**b**) definition of angles $\theta_{{\text{D}}}$, $\theta_{{\text{A}}}$, and $\theta_{{{\text{DA}}}}$, (**c**) orientation factors for 1224 structures of A647 generated by Euler angles, and (**d**) orientation factors larger than that of the MD structure. As shown in (**a**), new A647 structures were generated by rotating the structure *α* degrees around the *z* axis, then *β* degrees around the *y'* axis, and finally *γ* degrees around the *z''* axis. In (d), the real transition dipole moments of the top four representative results are shown in blue and superimposed on the MD result shown in red.

The above analysis confirms that the magnitude of the orientation factor for this system is much larger than the isotropic mean value. The results were obtained in a situation where A647 was bound to the linker, but various values of the orientation factor should be possible in situations where there are no constraints on the available angles due to the length of the linker. The increase in the orientation factor contributes to the enhancement of FRET efficiency. We therefore attempted to search for molecular orientations with a further larger value of the orientation factor under conditions free from the constraints of available angles due to linker length. For this purpose, Euler angles (*α*, *β*, *γ*), which is a useful method to specify rigid body rotation, were employed. A schematic illustration of Euler angles is shown in Fig. 5a. The rotation by Euler angles transforms the position ${\mathbf{r}}_{i} = \left( {x_{i} ,y_{i} ,z_{i} } \right)^{T}$ of the *i*-th atom to ${\mathbf{r^{\prime}}}_{i}$.

11 $${\mathbf{r^{\prime}}}_{i} = \left( {\begin{array}{*{20}c} {\cos \gamma } & { - \sin \gamma } & 0 \\ {\sin \gamma } & {\cos \gamma } & 0 \\ 0 & 0 & 1 \\ \end{array} } \right)\left( {\begin{array}{*{20}c} {\cos \beta } & 0 & {\sin \beta } \\ 0 & 1 & 0 \\ { - \sin \beta } & 0 & {\cos \beta } \\ \end{array} } \right)\left( {\begin{array}{*{20}c} {\cos \alpha } & { - \sin \alpha } & 0 \\ {\sin \alpha } & {\cos \alpha } & 0 \\ 0 & 0 & 1 \\ \end{array} } \right){\mathbf{r}}_{i} .$$

In﻿ the following, the given MD snapshot is coordinated as the starting structure (*α*, *β*, *γ*) = (0, 0, 0). The real transition dipole moment of the rotated molecule can be calculated by multiplying the transformed coordinates ${\mathbf{r^{\prime}}}_{i}$ by the transition charges $q_{i}$.

12 $${{\varvec{\upmu}}} = \sum\limits_{i} {q_{i} } {\mathbf{r^{\prime}}}_{i} .$$

Give﻿n the real transition dipole moments of the donor and acceptor, as shown in Fig. 5b, the three angles $\theta_{{\text{D}}}$, $\theta_{{\text{A}}}$, and $\theta_{{{\text{DA}}}}$ are determined. For example, $\theta_{{{\text{DA}}}}$ can be obtained as follows:

13 $$\theta_{{{\text{DA}}}} = \arccos \frac{{{{\varvec{\upmu}}}_{{\text{D}}} \cdot {{\varvec{\upmu}}}_{{\text{A}}} }}{{\left| {{{\varvec{\upmu}}}_{{\text{D}}} } \right|\left| {{{\varvec{\upmu}}}_{{\text{A}}} } \right|}}.$$

The analysis was performed using the following procedure. First, we isolated the A647 structure from the MD snapshot and generated its rotated structure data by fully varying Euler angles with a step of 20 degrees with the molecular center (the center of mass) as the origin. Next, out of the generated A647 structures, the ones overlapping with all protein (polypeptide) structures of LH2 were removed, and then the remainings were used to calculate the orientation factor. It should be noted that this analysis uses Euler angles to rotate A647, but not to translate it. Therefore, the intermolecular distance between A647 and BChl1 remains constant.

In total, 5832 structures of A647 were generated from the MD snapshot at 130 ns with various rotation angles. To consider collisions with the protein, A647 structures with a distance of less than 1 Å from the surrounding protein atoms were excluded from the generated structures. In this step, 4608 structures, corresponding to 79% of the total, were removed. This strongly suggests that the protein around A647 is spatially crowded, resulting in a significant deviation from the isotropic mean of the orientation factor. The remaining 1224 structures were used to calculate the orientation factor, and the results are shown in Fig. 5c. For a better understanding of the results in Fig. 5c, only values larger than the orientation factor ($\kappa^{2}$ = 1.72) for the MD structure (*α*, *β*, *γ*) = (0, 0, 0) are shown in Fig. 5d. The largest value of the orientation factor ($\kappa^{2}$ = 2.12) was obtained for (*α*, *β*, *γ*) = (100, 320, 100) and (280, 40, 280). From these results, we have successfully found A647 structures with a larger orientation factor than the starting MD structure. In addition, the present analysis shows that the orientation factor becomes large around *β* = 0 degrees, confirming that the A647 structure resulting from the MD simulation already has a large orientation factor.

As shown in Eq. (8), the orientation factor is defined using three vectors: the donor and acceptor real transition dipoles as well as the vector connecting them. To gain further insight into Fig. 5d, we analyzed the orientation of the real transition dipoles. Figure 5d also depicts the real transition dipoles of the top four representative A647 structures with prominent orientation factors. Here, the real transition dipoles of A647 in the MD structure are also superimposed. The results show that the real transition dipole for the A647 structure with the largest orientation factor is slightly deviated from that for the MD structure in terms of direction, while the real transition dipoles of the other three A647 structures are oriented similarly to the MD structure. Table 3 summarizes the values of the orientation factors and the angles constituting the orientation factors for these structures. It should be noted that the BChl1 structure in LH2 is fixed in this analysis, and therefore the angle $\theta_{{\text{A}}}$ is set as a constant ($\theta_{{\text{A}}}$ = 50 degrees). According to Eq. (8), it is expected that the magnitude of the orientation factor increases with an increase in $\cos \theta_{{\text{D}}}$. From this, we can see that the A647 structure with (*α*, *β*, *γ*) = (100, 320, 100) has an angle of $\theta_{{\text{D}}}$ = 13 degrees ($\cos \theta_{{\text{D}}}$ = 0.97), which is rather small and thus contributes significantly to the largest orientation factor. To further investigate the influence of $\theta_{{\text{D}}}$ on $\kappa^{2}$, the A647 structure with the angle $\theta_{{\text{D}}}$ close to zero, corresponding to (*α*, *β*, *γ*) = (240, 20, 280), was extracted from Fig. 5b and its orientation factor was determined. The resulting orientation factor was calculated to be 1.67, which was smaller than that of the MD structure ($\kappa^{2}$ = 1.72). This was attributed to the fact that although $\cos \theta_{{\text{D}}}$ takes a value close to 1, it largely cancels out $\cos \theta_{{{\text{DA}}}}$ (= 0.63) due to the difference of $\theta_{{{\text{DA}}}}$ (= 51 degrees) from 90 degrees. These results indicate that an appropriate combination of the two angle parameters, $\theta_{{{\text{DA}}}}$ and $\theta_{{\text{D}}}$, is necessary to increase the orientation factor for this system, and such a balanced set of angles was achieved for the A647 structure with the largest orientation factor.

**Table 3:** Angles of real transition dipole moments between A647 and BChl1 (degrees).

(*α*, *β*, *γ*)^a^ (degrees)	$\kappa^{2}$ ^b^	$\theta_{{{\text{DA}}}}$(degrees)	$\theta_{{\text{D}}}$(degrees)	$\theta_{{\text{A}}}$(degrees)
(0, 0, 0)	1.72	98	48	50
(100, 320, 100)	2.12	63	13	
(200, 20, 240)	2.00	81	33	
(340, 340, 60)	1.95	92	41	
(240, 0, 140)	1.80	94	45	
(240, 20, 280)	1.67	51	0.49	

^a^Values of Euler angles for the A647 structures shown in Fig. 5.

^b^Orientation factors obtained with the TrESP method (Eq. (10)).

## Conclusion

In this study, we investigated the orientation factor for the LH2-A647 conjugate via the computational approach with MD simulation and TrESP-based electronic coupling calculation. Our simulation predicted the value of the orientation factor $\kappa^{2}$ to be 1.55, which was much larger than the widely used isotropic mean value 2/3. The MD analysis confirmed that this large orientation factor is due to the linker bound to LH2, which greatly restricts the movement of A647. In addition, the orientation factor determined in this study was found to be clearly appropriate in the sense that its use for the determination of the A647-BChl distance in combination with the experimental parameters gave good agreement with the position of A647 fully based on the MD simulation, and differed significantly from the prediction based on the isotropic mean $\kappa^{2}$. These results strongly indicate that the isotropic mean value, which is commonly used in FRET analysis, is not suitable for the LH2-A647 system and that the orientation factor should be determined with explicit consideration of structural information to improve the accuracy of FRET analysis.

This study also examined the effects of spectral overlap and refractive index on the calculated intermolecular distance. The analysis revealed that in addition to the use of the above appropriate $\kappa^{2}$, the distance prediction based on the Förster theory can be further improved if the refractive index is smaller than the value used in the previous studies. The results also strongly suggested that the value of the refractive index used in the previous studies may not reflect the actual environment of the LH2-A647 system. On the other hand, this simulation-based analysis suggested that a spectral overlap four times larger than the previously reported value would be required to quantitatively reproduce the intermolecular distances, which is seemingly an unrealistic value. The results derived from our computer simulation will be useful to re-evaluate the values of these parameters for the validation of the FRET analysis. The Poisson-TrESP^71,72^ and polarizable continuum model (PCM)^73^ methods have been developed to accurately incorporate local field and screening effects on electronic coupling, which may lead to more accurate intermolecular distance calculations. On the other hand, Sobakinskaya et al. have shown that simply applying an effective dielectric constant to the TrESP method can successfully reproduce the electronic coupling results from the Poisson-TrESP method^32^. This fact may suggest that the use of a single refractive index employed in this paper is a reasonable approach. However, it should be interesting to use the Poisson-TrESP or PCM method in future work to calculate intermolecular distances with higher accuracy.

To further improve the FRET efficiency of the LH2-A647 system, we also searched for the optimal orientations of A647 with a large orientation factor using Euler angles. We considered the re-oriented structures of A647 having no overlapping with the protein. As a result of the analysis, we succeeded in finding several rotated A647 structures with a larger orientation factor than the MD structure. Such A647 structures may be realized artificially by changing the type and length of the linker connecting A647 and LH2. Experimental verification of our results is desired in the future.

In this study, we used Förster theory (Eqs. (1)–(4)) to analyze orientation factors and intermolecular distances. We focused our analysis on BChl1, which has the largest electronic coupling to A647, but the influence of BChls other than BChl1 in B850 can be considered using generalized Förster theory^74,75^ for more accurate analysis. The lack of consideration of generalized Förster theory is probably one of the main reasons why the intermolecular distances derived from Förster theory are not in quantitative agreement with the MD results. However, the time evolution of the intermolecular distances derived from the FRET rate is similar to the MD results in terms of relative trends (Fig. 3a), strongly suggesting that Förster theory is applicable in a qualitative sense to the estimation of intermolecular distances in this system. The calculation of intermolecular distances using generalized Förster theory is our future work.

Electronic coupling calculations using the TrESP method in combination with MD simulations and/or Euler angles are a promising approach for molecular orientation analysis. The TrESP method is based on the classical Coulomb interactions, and thus has a significant advantage in terms of computational cost and applicability compared to the TDFI and TDC methods, which are deeply based on quantum mechanical interactions. In addition, our previous studies have confirmed that the accuracy of the TrESP method is comparable to that of the TDFI method, except when the intermolecular distance is remarkably close^35^ or when the effect of transition quadrupoles is not negligible^36^. The features of this approach, such as low computational cost, high reliability, and wide applicability, will greatly contribute to the elucidation of molecular mechanisms involved in FRET phenomena and to the broadening of the scope of FRET analysis.

## Data Availability

The data that support the findings of this study are available from the corresponding author upon reasonable request.

## Abbreviations

FRET: Fluorescence resonance energy transfer
LH2: Light-harvesting 2 complex
A647: Alexa Fluor 647
MD: Molecular dynamics
BChl: Bacteriochlorophyll
TrESP: Transition charge from electrostatic potential
GFP: Green fluorescent protein
DD: Dipole–dipole
DA: Donor–acceptor
EET: Excitation-energy transfer
TDC: Transition density cube
TDFI: Transition-density-fragment interaction
LH1-RC: Light-harvesting 1-reaction center core complex
PDB: Protein data bank
POPE: 1-Palmitoyl-2-oleoyl-phosphatidylethanol-amine
RMSD: Root mean square deviation
PME: Particle mesh Ewald
GAFF: General Amber force field
TDDFT: Time-dependent density-functional theory
